# Cenderitide (CD-NP), a novel peptide designed to activate both guanylyl cyclase B and A, activates the second messenger cGMP, suppresses aldosterone, and preserves GFR without reducing blood pressure in a proof-of-concept study in patients with chronic heart failure

**DOI:** 10.1186/1471-2210-11-S1-P18

**Published:** 2011-08-01

**Authors:** Lisa C Costello-Boerrigter, John A Schirger, Wayne L Miller, Guido Boerrigter, Horng H Chen, Ruth Kempf, Candace Y Lee, John C Burnett

**Affiliations:** 1 Cardiorenal Research Laboratory, Mayo Clinic, Rochester, MN, USA

## Background

The development of new therapies for heart failure (HF) is a high priority. The endogenous natriuretic peptides (NPs) ANP, BNP, and CNP mediate cardiac unloading, renal enhancing, vasoprotective and RAAS suppressing actions via activation of the receptors guanylyl cyclase (GC)-A and GC-B, respectively. We engineered cenderitide (also known as CD-NP) as a novel NP that activates both GC-A and GC-B, with higher activity at GC-B so as to be less hypotensive than the GC-A agonists ANP and BNP. The cardiorenal and neurohumoral actions of cenderitide in HF patients are undefined. We hypothesized that cenderitide would suppress RAAS via GC-A activation, would unload the heart while maintaining blood pressure (BP) taking advantage of GC-B activation, and would preserve glomerular filtration rate (GFR) secondary to both GC-A and GC-B.

## Methods

Subjects with stable, chronic HF (ejection fraction ≤40%) were enrolled in a randomized, double-blind, placebo-controlled, proof-of-concept study. Randomization was to either placebo (n=5) or cenderitide (20 ng/kg/min; n=9; Nile Therapeutics) for 4 hours. On the study day, patients received their usual cardiac medications except diuretics. GFR was measured by iothalamate. BP, aldosterone and ANP were also assessed, the latter to assess cardiac unloading. After equilibration, a 60’ baseline clearance (C1) was done, followed by two 120’ clearances (C2 and C3, respectively) with cenderitide or placebo, and finally a 120’ post-infusion clearance (C4). Changes from baseline were compared between groups. p-values are cenderitide vs. placebo.

## Results

Cenderitide increased cGMP, the second messenger of the NPs, in C2 and C3 (p<0.05). Cenderitide as compared to placebo did not change BP or heart rate. GFR (p=0.04) increased with cenderitide in C2. Cenderitide decreased aldosterone in C3 (p=0.01) and tended to do so in C2 (p=0.054). Cenderitide decreased ANP in C4, consistent with greater cardiac unloading.

## Conclusion

Cenderitide represents a novel dual activator of GC-A and GC-B which in human HF is safe, activates cGMP, suppresses aldosterone, unloads the heart and preserves renal function at a non-hypotensive dose. Further investigations of this promising new therapeutic strategy for HF are warranted.

